# An Assessment of Dietary Exposure to Cadmium in Residents of Guangzhou, China

**DOI:** 10.3390/ijerph15030556

**Published:** 2018-03-20

**Authors:** Weiwei Zhang, Yungang Liu, Yufei Liu, Boheng Liang, Hongwei Zhou, Yingyue Li, Yuhua Zhang, Jie Huang, Chao Yu, Kuncai Chen

**Affiliations:** 1 Guangzhou Center for Disease Control and Prevention, Guangzhou 510440, China; bin_cheng818@163.com (W.Z.); gzliuyufei@hotmail.com (Y.L.); liangboheng_1999@163.com (B.L.); gzcdczhw@126.com (H.Z.); gzcdclyy@163.com (Y.L.); pisceszyh@126.com (Y.Z.); huangjie1026@126.com (J.H.); yuchaogzcdc@163.com (C.Y.); 2 Department of Toxicology, School of Public Health, Southern Medical University, Guangzhou 510515, China; yungliu@fimmu.com

**Keywords:** food, cadmium, dietary exposure assessment

## Abstract

Cadmium and its compounds are human carcinogens with severe organ toxicity, and their contamination of agricultural soil in China has been frequently reported; however, the dietary exposure to cadmium in residents and the relevant health risk have seldom been reported. In this study, the concentration of cadmium in various types of food collected from 2013 to 2015 were analyzed using graphite furnace atomic absorption spectrometry, and the dietary exposure to cadmium assessed based on a dietary survey in 2976 Guangzhou residents. In total, 3074 out of 4039 food samples had cadmium levels above the limit of detection. The mean ± standard deviation (50th, 95th percentile) cadmium content in all samples was 159.0 ± 112.7 (8.6, 392.4) μg/kg, with levels ranging from 1.0 to 7830 μg/kg. Using the mean cadmium concentrations, the average monthly dietary exposure of Guangzhou residents to cadmium was 14.4 (μg/kg body weight (BW), accounting for 57.6% of the provisional tolerable monthly intake (PTMI). Rice, laver, vegetables, and live aquatic products were the main sources of cadmium intake, on average accounting for 89% of the total value. The dietary cadmium exposure in high consumers (95th percentile food consumption) was 41.0 μg/kg·BW/month, accounting for 163% of the PTMI. Additionally, dietary cadmium exposure at mean consumption but high cadmium food concentration (95th percentile) was 32.3 μg/kg·BW/month, corresponding to 129% of the PTMI. The level of dietary exposure to cadmium in most Guangzhou residents was within the safety limit, thus increased health risk from dietary cadmium exposure is low at present. However, continued efforts by local governments to monitor the levels of cadmium in the four main food categories contributing to exposure are necessary.

## 1. Introduction

Cadmium has been widely discharged into the environment through industrial emission, waste incineration, and other ways, in some rapidly developing countries and regions, such as China, in the past 30 years. Cadmium occurs in some industrial processes and the environment in the form of either an elemental substance (metal) or various compounds (e.g., cadmium sulfate, chloride, and oxides). Even in areas remote from cadmium emission, such as farmlands without industrial pollution, cadmium can also accumulate through atmospheric deposition and the use of Cadmium-containing fertilizers [1]. The levels of contamination of agricultural soil vary greatly in different regions/areas. Particularly, there is great concern regarding human exposure to cadmium in some regions in China. For example, in the vast Pearl River Delta region of South China, activities of cadmium-discharging industries have been existed since 1980s. Diet is the major source of cadmium exposure [2]. The levels of cadmium in foods vary greatly, depending on both the concentration of cadmium in the agricultural soils in different areas and the category of agricultural/aquatic products. Cadmium can be taken up by crops and aquatic plants such as rice, vegetables, and seaweed, or be absorbed by livestock/poultry/aquatic animals and accumulated in the gastrointestinal systems. Thus, the level of cadmium concentration in crops, in the internal organs of livestock/poultry, and in aquatic animals and plants (such as shellfish, fish, and seaweed), is proportional to cadmium concentrations in the environment [3]. Historically, cadmium caused the infamous itai-itai disease in the 20th century in Japan (Toyama Prefecture), which caused softening of the bones, severe pain, and kidney failure [4]. Though new typical cases of this disease have not been reported for decades, a few studies suggest that the dietary exposure to cadmium through food is currently high enough to pose a potential hazard to human health in many areas [5]. Recent data on risk assessment of cadmium exposure in human populations indicates increased risk of cancer in relation to cadmium exposure even at low levels [6,7,8], with females being more susceptible [9].

Toxicological studies on cadmium suggest that its exposure may cause increased genomic instability [10] and other epigenetic effects in humans, endpoints commonly recognized as mechanisms of carcinogenesis [11]. Evidence shows that exposure to cadmium is positively associated with cancer in the kidneys and prostate [12]. Cadmium and its compounds have been classified by the International Agency for Research on Cancer as group 1 (human) carcinogens, based on sufficient evidence for their carcinogenicity in animals and induction of cancers in humans [12]. According to the Integrated Risk Information System provided by the U.S. Environmental Protection Agency in 2015, chronic oral exposure of humans to cadmium results in a build-up of cadmium in the kidneys that can cause kidney diseases [13]. Cadmium-associated kidney disorders include kidney stone formation and proteinuria [14]. In the liver, cadmium may bind to metallothionine, and the bound form of cadmium may spread through the blood flow to various organs/tissues; in particular, it may be selectively accumulated in the kidneys [15]. Renal tubular damage was observed at lower cadmium exposure than anticipated in previous studies [16], and it is now acknowledged that renal dysfunction is the most sensitive toxicological endpoint arising from cadmium exposure [17].

In fast-developing countries such as China, contamination of the environment with cadmium has become a common and serious public concern [18,19]. Following the collapse of a tailings dam near a lead and zinc mine in Chenzhou, Hunan Province, China in 2008, mining waste spilled and spread to the farmlands situated along the Dong River and led to high contamination of rice with cadmium [20].

Dietary exposure to cadmium and its compounds through consumption of grains and aquatic products has been estimated to be prominent in people living in South China [21,22,23]. This has raised public concern about the possibility of human health effects due to some biological changes observed in a population heavily exposed to cadmium which was released from illegal waste disassembling activities in some areas in China [18,19,24]. The large variation in cadmium exposure between different regions in China is influenced not only by cadmium in the environment, but also with differences in food types and dietary habits.

Guangzhou is the capital and largest city of Guangdong province; it is also home to 12.78 million people. Guangzhou is neither a heavy industrial nor a mineral city. According to a preliminary risk assessment of the dietary exposure to cadmium in Guangdong, results showed that average dietary cadmium exposure from the main food did not exceed the PTMI [25]. However, as far as we know, few data on the dietary exposure to cadmium have been reported in Guangzhou. Based on the high consumption of rice in southern China and a relatively high consumption of seafood in Guangzhou, it is meaningful to evaluate the dietary cadmium exposure and the relevant health risk in residents of Guangzhou City. The aim of this study was to evaluate dietary intake of cadmium and the relevant health risk in residents of Guangzhou City.

## 2. Materials and Methods 

### 2.1. Food Sampling

For this study, investigators purchased food samples from retail shops that covered 12 districts of Guangzhou City during 2013–2015. Three streets (two central and one remote) were randomly selected in each district as the monitoring sites. At each monitoring site, retailer selection included supermarkets, agricultural wholesale markets, shops, and restaurants; those were selling foods that fall into the monitoring category. Food categories were included grains (rice, wheat, millet, corn); live aquatic products (fish, shrimp, shellfish, crabs); meat (livestock meat, liver, and kidney); poultry, eggs, and milk (liquid); vegetables, fruit, fungi, and algae (dry mushrooms and laver); beans (mung bean, soybean); drinks (juice); and water. A total of 4039 single-species food samples were collected. Samples were kept in sealed plastic bags and transferred to the laboratory as soon as possible. Samples were homogenized immediately and stored frozen until digestion.

### 2.2. Analytical Procedure (Atomic Absorption Spectrophotometry)

The procedure to determine cadmium in foods was in accordance with the method previously reported [26] with slight modifications. A quantity of 300–500 mg of each sample was placed in a digestion tube, to which was then added 5 mL of concentrated nitric acid and 2 mL of 30% hydrogen peroxide, and the mixture was held for 1.5–2.0 h. At the same time, blank and reference tests were also prepared. Cadmium content was determined by graphite furnace atomic absorption spectrophotometry, detected with a PinAAcle 900T (G) (PerkinElmer, Beijing, China) atomic absorption spectrophotometer and a deuterium lamp, with the background signal corrected by a self-absorption correction method.

### 2.3. Quality Control

The cadmium reference materials were certified as heavy metals and granted certificates by the Chinese scientific community. Nitric acid and hydrogen peroxide used in this study were of ultrapure grade. Ultrapure water (with a resistivity of 18.2 MΩcm) was produced by a Millipore system (Kurita Water Industries, Carrollton, TX, USA), and purified water was purchased from Wahaha Corp (Hangzhou, China). National first-level standard material (GBW10035) cadmium, cadmium standard in wheat powder (purchased from Chinese Academy of Geographical Sciences, Beijing, China), 74 ± 3 μg × kg^−1^ for cadmium, was used as a reference (quality control) for detection of cadmium in the samples. The limit of detection (LOD) for elemental cadmium was 1 μg × kg^−1^, and the limit of quantification was 3 μg × kg^−1^, with an accuracy of <5% deviation from the real values.

### 2.4. Estimation of Daily Food Consumption

Food consumption amounts in the relevant population were calculated from a food consumption survey of urban and rural residents in Guangzhou City in 2011 (as published in a Chinese journal) [27]. In the survey, information was obtained on all individuals who received questionnaires at home. The questionnaire contained basic demographic information (including age, gender, job, etc.) and dietary consumption information. For children younger than 7 years and for elderly persons 75 years or older, the questionnaires were completed by their adult family members. Food intake data was obtained using a 3-day, 24-h recall (i.e., 3 days’ retrospective food record) to reflect daily consumption as precisely as possible. Two weekdays in combination with Saturday or Sunday were included in the investigation. The food was classified according to the food composition table [28].

Food consumption was expressed as the amount consumed by a reference person per day derived from individual dietary consumption data (C). Dietary consumption for a reference person per day (Cr), based on age, gender, labor intensity, and physical wellness status, is commonly used in dietary research and may be applied to assess the overall food or nutrient intake in a population [29,30]. The formula is Cr = (e) × C/3. An individual’s reference coefficient (e) is defined as the ratio of individual estimated energy requirement with reference to an 18-year-old male with light physical activity. According to Chinese dietary reference intakes [31], the estimated energy requirement for an 18-year-old male weighing 65 kilograms with slight physical activity is 2250 kcal per day. Therefore, (e) is equal to the individual estimated energy requirement/2250.

### 2.5. Exposure Assessment

Mean monthly exposure to cadmium was estimated as the average amount of food consumed per kg body weight (BW) based on thee recorded days and multiplied by the mean concentration of cadmium in each food group. High monthly exposure (95th percentile food intake) was estimated using mean cadmium concentrations in each food group combined with 95th percentile food consumption. Additionally, high monthly exposure was estimated using average food consumption multiplied by 95th percentile food concentrations (Supplemental Table S1). The estimated monthly exposure was compared with the provisional tolerable monthly intake (PTMI) of 0.025 mg/kg BW (equivalent to 25 μg/kg BW) established by the Joint Food and Agriculture Organization of the United Nations/World Health Organization Expert Committee on Food Additives in 2010 [32].

The monthly dietary exposure to cadmium per kg BW was calculated using the equation

$$\mathrm{Exp}=\overset{n}{\underset{i=1}{\sum }}=\frac{\mathrm{Fi}*\mathrm{Ci}}{W}*30$$

Exp is the monthly dietary cadmium intake per kg BW (mg/kg·BW/month), Fi is the daily consumption (mean daily consumption for mean exposure, P95 daily consumption for high exposure) of each food category for reference person (g/person/day), Ci is the mean concentration of cadmium in each sample of each food category (mg/kg), and W is the body weight of each respondent (kg). When a Ci value was lower than LOD, it was then assumed to be LOD/2.

## 3. Results

### 3.1. Distributions of Age and Gender in the Study Population

A total of 998 households, with 2976 residents who were more than 3 years old, were included in the study. Among the subjects, 1422 were male (48%) and 1554 were female (52%). The age range of the subjects was 3–88 years old, and the average age was 31.9 years. The age group of 3–5 years old accounted for 8% (223), 6–17 years old accounted for 21% (622), 18–49 years old accounted for 59% (1759), and 50 years old and above accounted for 12% (372). The subjects were students or preschool children (835, 28%), employed (1828, 61%), and unemployed (313, 11%). Among the employed subjects, 1356 did light physical labor (e.g., teachers, civil servants, salesmen, etc.), 339 did moderate physical labor (e.g., factory workers, cleaners), and 133 did heavy physical labor (e.g., porters).

### 3.2. Cadmium Concentration in Each Category of Food and Water

The measured concentrations of cadmium in 4039 food samples obtained during 2013–2015 are summarized in Table 1. The average cadmium content in all samples was 159 μg/kg, and the 50th percentile (P50) and 95th percentile (P95) values were 8.6 μg/kg and 392 μg/kg, respectively. In total, 76.1% (3074 of 4039) of the samples had cadmium levels above the LOD. Among all food categories in this study, laver (dry) had the highest concentration of cadmium, with the detected values ranging from not detected (ND) to 4960 μg/kg, and a mean of 1871 μg/kg. Shellfish ranked second highest, with cadmium values ranging from 1.0–7830 μg/kg and a mean of 1665 μg/kg. In water, eggs, milk, fruit juices, and poultry, cadmium concentrations were very low, with most values being lower than the detection limit.

### 3.3. Dietary Cadmium Exposure

The average and 95th percentile food consumption in 2976 Guangzhou residents are reported in Table 2. Our results show that vegetables was the food category consumed most abundantly, with a mean of 235 g/day. Rice was the second largest category, with a mean consumption of 152 g/day. Cadmium exposure was assessed by applying the equation presented at the end of Section 2.5. The average dietary exposure to cadmium through consumption of the 11 food categories was 14.4 μg/kg·BW/month based on mean cadmium food concentrations. This corresponds to 57.6% of the PTMI (Table 2). Cadmium exposure at P95 consumption was 41.0 μg/kg·BW/month corresponding to 163% of the PTMI. Additional analysis of high exposure by using mean consumption combined with P95 cadmium concentrations in food was 32.3 µg/kg·BW/month, corresponding to 129% of the PTMI (Supplemental Table S1).

Of the 11 food categories, rice, laver, vegetables, and live aquatic products were the main sources of cadmium exposure, and these four types of food accounted for 89.1% of the total dietary exposure to cadmium, of which rice was the major contributor (46.4%) of cadmium in the Guangzhou population. Categories of food other than those four contributed about 10% to the total dietary cadmium exposure. These results indicate that the increased health risk of dietary cadmium exposure was low in most of the Guangzhou population; however, for individuals who consume large amounts of food (especially the four major contributors to cadmium exposure), health risk from dietary cadmium exposure cannot be excluded.

## 4. Discussion

### 4.1. Relatively High Levels of Cadmium in Some Categories of Food

As indicated in Table 1, the concentration of cadmium in 4039 food samples demonstrated a wide range, with large differences between various categories of food, among which aquatic products showed the highest concentrations, followed by livestock kidney. In most other food categories—such as egg, milk, and poultry meat—cadmium levels were either under the limit of detection or very low. As indicated in Table 1, the mean concentration of cadmium in rice, the dominant energy source for residents in South China, was 88.0 μg/kg, which was slightly higher than the concentrations reported in Guangdong Province (77.0 μg/kg) [25] and Shanghai City (71.0 μg/kg) [33], and lower than the level in Guangxi Province (127.0 μg/kg) [34].

### 4.2. Levels of Dietary Cadmium Exposure in Residents of Guangzhou

As indicated in Table 2, the monthly dietary cadmium exposure in residents of Guangzhou was lower by about half than the average JECFA (Joint Expert Committee on Food Additives) of PTMI, and this suggests low average health risk of dietary cadmium exposure in Guangzhou residents. This conclusion is consistent with the relevant assessment results obtained in Shenzhen [35], a large city near Guangzhou and Shanghai [33] located in eastern China. The main dietary sources of cadmium in Guangzhou residents are rice, laver, vegetables, and aquatic products, in decreasing order (see Table 2). This pattern of food consumption is different from that of residents of some other cities in China, e.g., the consumption of various foods for residents of Shanghai is in the order of vegetables, rice, and aquatic products [33], while for residents of Jinan it is aquatic products, wheat, and vegetables [36]. It should be noted that in northern China, people prefer wheat products (e.g., noodles and steamed bread) rather than rice, and the cadmium level in wheat is apparently lower than in rice, as indicated in Table 1. Additionally, most areas in northern China are remote from seashores, and therefore seafood is relatively unavailable, expensive, and rarely consumed; this might further decrease exposure to cadmium in the inner and northern areas of China. In the National Diet and Nutrition Survey conducted in 2002, the consumption of laver was not reported. However, unlike other parts of China, laver is a popular food in the Guangdong province, especially in coastal areas. Therefore, the consumption of laver was listed separately in this study (Table 2). Our results demonstrated that laver makes a substantial contribution to cadmium exposure. To sum up, most Guangzhou residents have dietary exposure to cadmium within the safety limit, while some groups of the population—i.e., individuals either with very high food consumption (P95) or individual exposed to cadmium at very high (P95) concentrations—exceeded the PTMI and may be at increased risk of adverse health effects. 

### 4.3. Limitations of the Study 

There are indeed some limitations of the current study. First, this study was a point assessment for dietary exposure, which may bias the estimated the cadmium exposure even though we aimed to include all major food categories in the foods sampled for determination of cadmium concentrations. Second, only three-day food records were used to assess consumption data. The short time frame may not reflect the eating habits of the whole year. Therefore, increasing the number of recording days and repeated assessment during different seasons may provide a more accurate assessment of habitual food consumption. Third, the standard of LOD of 1 mg/kg used in this study appears to be high but was consistent with Chinese National Food Contamination Monitoring Program. The higher LOD may overestimate the average and high monthly dietary exposure of Guangzhou residents to cadmium.

## 5. Conclusions

It might be true that residents living in South China, especially areas near the seashore, have a relatively greater chance of increased cadmium exposure. However, as estimated in the present study, increased health risk from dietary cadmium exposure is low in the majority of the residents in Guangzhou. However, individuals with high consumption (above the 95 percentile) of the food items contributing most to cadmium exposure exceed PTMI both at mean and P95 cadmium concentration, and this should be further investigated. This study shows that consumption of rice, laver, vegetables, and live aquatic products by Guangzhou residents contributes with about 90% of the total dietary cadmium exposure. This provides a clear clue for appropriate health education delivered to the general public: consumption of foods other than the above four should be encouraged—e.g., consumption of more wheat products and less rice, and more livestock and poultry (milk or meat, not kidney)—may reduce cadmium exposure. Furthermore, continued efforts by local governments to monitor the concentration of cadmium in those four food categories contributing to exposure are necessary. 

## Figures and Tables

**Table 1:** Cadmium concentrations in food and water in Guangzhou City from 2013 to 2015.

Food Category	N	<LOD	Cadmium Concentration (μg/kg)			
			Mean ± Standard Deviation	P50	P95	Range
Rice	454	36	88.0 ± 8.5	83.0	170	2.6–440
Wheat	618	53	16.6 ± 5.9	13.9	32.0	6.0–117
Millet	94	0	24.8 ± 4.7	16.7	55.7	2.4–90.7
Corn	327	46	10.1 ± 9.5	2.7	31.8	1.1–99.6
Other corns (coarse cereals)	294	82	56.3 ± 19.2	24.0	144	6.0–330
Fish	298	197	8.7 ± 17.9	ND	31.0	6.0–275
Shrimp	118	26	14.7 ± 39.2	3.3	19.6	7.0–378
Shellfish (bivalves and only meat; visceral organs removed)	97	7	1665 ± 1105	876	4300	29.0–7830
Crab (only white claw meat)	11	0	815 ± 740	282	1640	16.0–5550
Livestock meat	289	56	5.3 ± 7.1	3.7	35.0	2.4–90.0
Liver	122	3	39.1 ± 18.3	31.0	71.6	5.0–239
Kidney	28	3	384 ± 399	167.5	509	8.0–4340
Poultry	26	21	0.6 ± 0.5	ND	1.1	1.0–1.3
Egg	85	77	0.9 ± 2.2	ND	3.0	1.0–4.0
Milk	20	20	0.5 ± 0.0	ND	ND	ND
Fruit juice	62	22	2.7 ± 3.9	2.0	5.4	1.0–12.0
Soybean	110	12	18.1 ± 11.3	16.0	40.0	1.0–80.0
Vegetable	450	37	18.7 ± 38.1	11.0	50.0	1.6–334
Fruit	40	9	4.2 ± 3.2	4.0	7.1	2.0–26.0
Mushroom	48	20	10.8 ± 8.6	5.0	27.0	2.0–42.0
Laver	210	0	1871 ± 1990	2119.1	3746	3.0–4960
Water	238	238	0.5 ^a^ ± 0.0	ND	ND	ND
Mean	4039	965	159 ± 113	8.6	392	1.0–7830

ND: not determined as all samples were <LOD. LOD: limit of detection ^a^ Unit of measurement in water is μg/L.

**Table 2:** Dietary cadmium (Cd) exposure in Guangzhou residents.

Food Category	Food List	Mean Exposure *			P95 Exposure **		
		Mean Dietary Consumption Reference Person (g/Day)	Cd Exposure (μg/Day)	Contribution (%)	P95 Dietary Consumption Reference Person (g/day)	Cd Exposure (μg/Day)	Contribution (%)
Crops	Rice	152	13.4	46.4	375	33.0	40.3
	Wheat	46	0.8	2.8	150	2.5	3.1
	Millet	8.9	0.2	0.7	67	1.7	2.1
	Corn	6.6	0.07	0.2	38	0.4	0.5
	Others	0.5	0.03	0.1	1.7	0.09	0.1
Aquatic food	Fish	43	0.2	0.7	126	0.5	0.6
	Shrimp	5.2	0.08	0.3	34	0.5	0.6
	Shellfish	1.2	2.0	6.9	1.7	2.8	3.4
	Crab	1.0	0.8	2.8	3.5	2.9	3.5
Meat	Livestock meat	121	0.7	2.4	324	1.9	2.3
	Liver	0.2	0.01	0.0	5.6	0.2	0.2
	Kidney	0.2	0.06	0.2	6.7	2.6	3.2
Poultry	Poultry	110	0.08	0.3	161	0.1	0.1
Egg	Egg	31	0.03	0.1	85	0.08	0.1
Milk	Milk	53	0.01	0.0	233	0.06	0.1
Drink	Fruit juice	0.9	0.01	0.0	17	0.05	0.1
Bean	Soybean	12	0.2	0.7	65	1.2	1.5
Vegetable	Vegetables	235	3.8	13.2	666	10.3	12.6
Fruit	Fruits	45	0.1	0.3	265	0.5	0.6
Fungi and algae	Mushroom	8.7	0.09	0.3	44	0.5	0.6
	Laver	3.0	5.6	19.4	10.	18.7	22.8
Water	Water	1.2 (L)	0.6	2.1	2.6 (L)	1.3	1.6
Daily total intake (μg/day)		28.9			81.9		
Total intake per month (μg/kg·BW/month)		14.4			41.0		
Contribution to PTMI (%)		57.6			163		

* Mean exposure was estimated using the average amount of food consumed per kg body weight (BW) based on three recorded days multiplied by the mean concentration of cadmium in each food group. ** P95 exposure was estimated using the 95th percentile food consumption multiplied by the mean concentration of cadmium in each food group. PTMI: provisional tolerable monthly intake. (L) means liter.

## Supplementary Materials

The following are available online at http://www.mdpi.com/1660-4601/15/3/556/s1, Table S1: Dietary cadmium exposure supplementary.
