# Supplementary material for: An Assessment of Dietary Exposure to Cadmium in Residents of Guangzhou, China
Source: Int J Environ Res Public Health. 2018 Mar 20;15(3):556. doi: 10.3390/ijerph15030556 (PMC5877101; doi:10.3390/ijerph15030556)
Supplement: Supplementary file 1 [file ijerph-15-00556-s001.pdf]

supplementary analysis on distribution of Cd exposure

Table S1. Dietary cadmium exposure supplementary

Food category	Food list	Mean dietary consumption reference person/day (g)	Cadmium level（μg/kg）	
			P50-Cd exposure per day（μg/d）	P95-Cd exposure per day（μg/d）
Crops	Rice	152	12.6	25.8
	Wheat	46	0.6	1.5
	Millet	8.9	0.2	0.5
	Corn	6.6	0.02	0.2
	Others	0.5	0.01	0.06
Aquatic food	Fish	43	—[a]	1.4
	Shrimp	5.2	0.02	0.1
	Shellfish	1.2	1.0	5.0
	Crab	1.0	0.3	1.6
Meat	Livestock meat	121	0.5	4.3
	Liver	0.2	0.00	0.01
	Kidney	0.2	0.03	0.08
Poultry	Poultry	110	—	0.1
Egg	Egg	31	—	0.09
Milk	Milk	53	—	—
Drink	Fruit juice	0.9	0.00	0.00
Bean	Soybean	12	0.2	0.5
Vegetable	Vegetables	235	2.5	11.7
Fruit	Fruits	45	0.2	0.3
Fungi and algae	Mushroom	8.7	0.04	0.2
	Laver	3.0	6.4	11.3
Water	Water	1.2 (L)	—	—
Daily total intake（μg/d）		/	24.6	64.7
Total intake per month（μg/kg BW/month）		/	12.3	32.3
Contribution to PTMI（%）		/	49.2	129

a: ND combined with mean dietary consumption reference person/day
